# Molecular Characterization and Disease Control of Stem Canker on Royal Poinciana (*Delonix regia*) Caused by *Neoscytalidium dimidiatum* in the United Arab Emirates

**DOI:** 10.3390/ijms21031033

**Published:** 2020-02-04

**Authors:** Seham M. Al Raish, Esam Eldin Saeed, Arjun Sham, Khulood Alblooshi, Khaled A. El-Tarabily, Synan F. AbuQamar

**Affiliations:** 1 Department of Biology, College of Science, United Arab Emirates University, Al-Ain 15551, UAE; 200440261@uaeu.ac.ae (S.M.A.R.); arjunsham@uaeu.ac.ae (A.S.); 201404120@uaeu.ac.ae (K.A.); 2 Khalifa Center for Genetic Engineering and Biotechnology, United Arab Emirates University, Al-Ain 15551, UAE; esameldin_saeed@uaeu.ac.ae; 3 College of Science, Health, Engineering and Education, Murdoch University, Murdoch, WA 6150, Australia

**Keywords:** chemical fungicide, disease control, *Neoscytalidium dimidiatum*, royal poinciana, stem canker, UAE

## Abstract

In the United Arab Emirates (UAE), royal poinciana (*Delonix regia*) trees suffer from stem canker disease. Symptoms of stem canker can be characterized by branch and leaf dryness, bark lesions, discoloration of xylem tissues, longitudinal wood necrosis and extensive gumming. General dieback signs were also observed leading to complete defoliation of leaves and ultimately death of trees in advanced stages. The fungus, *Neoscytalidium dimidiatum* DSM 109897, was consistently recovered from diseased royal poinciana tissues; this was confirmed by the molecular, structural and morphological studies. Phylogenetic analyses of the *translation elongation factor 1-a* (*TEF1-α*) of *N. dimidiatum* from the UAE with reference specimens of Botryosphaeriaceae family validated the identity of the pathogen. To manage the disease, the chemical fungicides, Protifert^®^, Cidely^®^ Top and Amistrar^®^ Top, significantly inhibited mycelial growth and reduced conidial numbers of *N. dimidiatum* in laboratory and greenhouse experiments. The described “apple bioassay” is an innovative approach that can be useful when performing fungicide treatment studies. Under field conditions, Cidely^®^ Top proved to be the most effective fungicide against *N. dimidiatum* among all tested treatments. Our data suggest that the causal agent of stem canker disease on royal poinciana in the UAE is *N. dimidiatum*.

## 1. Introduction

Royal poinciana (*Delonix regia* (Bojer ex Hook.) Raf.) is a beautiful flowering and shady branching tree. This member of the pea family (Fabaceae), which is also known as flamboyant, peacock or flame tree, can be recognized by the color of flowering cultivars, ranging from deep red to bright orange or yellow [1]. It is a rapid growing tree that can reach to 6–12 m height, and bears compound leaves that reach 30–60 cm length and flat woody pod fruits of about 60 cm long [2]. Despite it is native to Madagascar and tropical regions, this deciduous tree provides landscape with cooling shade during hot summers and warming-sunshine winters. In addition to the “umbrella” canopy it provides, royal poinciana can grow in a variety of soil conditions, and is highly tolerant to drought and salinity [3]. For that reason, there is a growing interest in the plantations of royal poinciana in the United Arab Emirates (UAE), mainly in parks, sidewalks, streets, parking lots and open areas. Although, this tree does not often suffer from real problems, stem canker has currently become a serious disease affecting royal poinciana. Therefore, it is urgent to address this present threat to royal poinciana in the UAE and worldwide.

Like other ornamental and stone fruit trees, fungi can attack different parts or tissues of royal poinciana under certain favorable conditions to cause canker diseases [3,4]. In general, cankers are destructive diseases which may cause damage to the whole or parts of trees such as branches, barks and woods. Fungi such as *Nectria galligena, Leptosphaeria maculans, Lasiodiplodia theobromae* and *Teratospheria zuluensis* are among those associated with canker diseases on sweet birch tree (*Betula lenta*), oilseed rape (*Brassica napus*), eucalypt and pine trees [5,6,7,8]. *Neoscytalidium dimidiatum* is another fungal pathogen that causes cankers and has a wide geographical and host range, including plum, almond (*Prunus dulcis*), mango (*Mangifera indica*), pitahaya (*Hylocereus undatus*), Citrus, Musa, Populus, and Ficus spp. in Australia, China, Egypt, Niger, Tunisia and the USA [9,10,11,12,13,14,15].

In Oman, stem canker has been reported on different trees including royal poinciana [16]. Symptoms can be recognized as branch wilt, dieback, canker, gummosis and death of infected trees. In general, severity of the disease caused by this fungus can be enhanced by stress factors such as water stress [9,16]. In the UAE, recent studies on tree diseases caused by fungi have reported black scorch disease and sudden decline syndrome (SDS) on date palm, and dieback disease on mango caused by *Thielaviopsis punctulata*, *Fusarium solani* and *L. theobromae*, respectively [17,18,19]. So far, there are no reports about royal poinciana-*N. dimidiatum* interaction causing stem canker disease in the UAE. 

Plant disease management mainly relies on the life cycle of the pathogen. *N. dimidiatum* produces two types of spores, pycniospores which are formed in pycnidia embedded in mature lesions and phragmospores which are formed by the breaking up of individual or groups of cells of mature hyphae in dead tissues of the lesion [20,21]. In culture, only phragmospores are formed and produced. Typically, cultural and horticultural practices such as pruning and fertilization may lower the risk of the pathogen, increase the vigor of the tree and extend its life [3]. On the other hand, such practices can be harmful due to the improper timing, unsterile tools, inexperienced persons or advanced stages of the pathogen’s life cycle. Regardless of its ecological problems and human health concerns, the use of chemical fungicides is yet the main disease management tactic to attenuate the threat of crop diseases [17,18,19,22]. In vitro treatment with the chemical, Beltanol-L (8-hydroxyquinoline), effectively inhibited the growth of *N. dimidiatum* in vitro [23]. The same fungicide also reduced symptoms of canker lesions on the seedlings of *Eucalyptus camaldulensis* under greenhouse conditions. Application of any of the systemic fungicides, Elsa^®^ (carbendizim), Mizab^®^ (mancozeb) or Curzate^®^ (cymoxamil), showed a significant inhibition to this fungus that causes wilt and canker diseases on cypress trees [24]. Hence, one should take into consideration the timing for minimum effective dose of the fungicide application to control the disease. 

Our long-term goal is to develop and implement integrated disease management (IDM) strategies using a combination of cultural, chemical and biological control with resistant cultivars of royal poinciana to manage stem canker disease. In the present investigation, an attempt was made to explore the feasibility of using efficient chemical fungicide(s) for the management of stem canker of royal poinciana. Therefore, our objectives were to: (1) isolate and identify the pathogen associated with infected plants; (2) evaluate the efficacy of fungicides against the causal agent of stem canker *in vitro*; (3) assess the potential fungicides against the pathogen in vivo under greenhouse conditions; and (4) manage disease of naturally infested plants in the field using the proper fungicide treatment. Here, we reported the assessment of systemic chemical fungicide treatments against *N. dimidiatum in vitro*, in the greenhouse as well as in the field. We also developed a short-term strategy to reduce the economic losses associated with stem canker disease. Future directions to employ research on biological control agents (BCAs) to suppress the damaging activities of the pathogen and to lower the risk of the disease on royal poinciana will further cooperate in the development of effective IDM programs.

## 2. Results

### 2.1. Symptoms of Stem Canker Disease on Royal Poinciana

**Figure 1:**
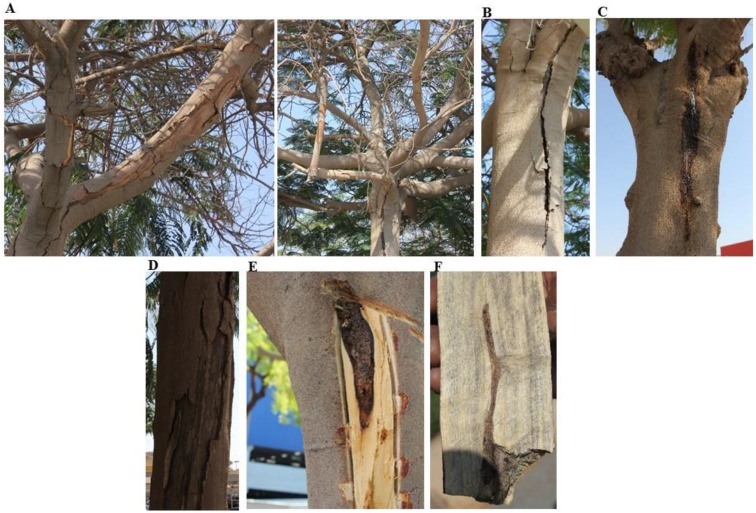
Symptoms of stem canker on trees of royal poinciana. (**A**) Severe symptoms of canker (left) and dieback (right); (**B**) typical longitudinal canker symptoms on stem; (**C**) gumming symptoms of the disease on the bark with fungal growth apparent beneath periderm; (**D**) main stem with the black stromata where the periderm has peeled away; (**E**) canker associated with internal symptoms in the trunk; and (**F**) affected vascular tissues. In (**A–F**), naturally infested royal poinciana trees with *N. dimidiatum* in DFC, UAE.

Disease symptoms of stem/branch cankers associated with dieback were observed in the orchard of royal poinciana distributed in Dubai Festival City (DFC), UAE (Figure 1A). Apparently, the pathogen was able to attack different tissues of royal poinciana, and the trees were severely affected leading to progressive dieback. In general, cankers on branches were detected in young trees. Stem cankers were observed in old and mature trees, and were associated with pruning wounds and other wounds (Figure 1A).

Cankers were developed longitudinally (Figure 1B), causing dark discoloration of xylem tissues and extensive gumming (Figure 1C). The main stem was often associated with black stromata, resulting the epidermis to peel away (Figure 1D). The discoloration continued outward, rotting symptoms led to spur and shoot blight was also observed. Sap was initially amber in color but later became dark. Internally, canker (Figure 1E) and affected vascular tissues (Figure 1F) were associated with this disease. Eventually, all royal poinciana trees were simultaneously found infected in the orchard (Figure 1A). These signs on royal poinciana are typical of stem canker that is known to be caused by a soil-borne wound pathogen. Therefore, attempts to isolate the putative pathogen from diseased royal poinciana was the first step in identifying the causal agent of this disease.

### 2.2. Identification and Molecular Characterization of Neoscytalidium Dimidiatum

**Figure 2:**
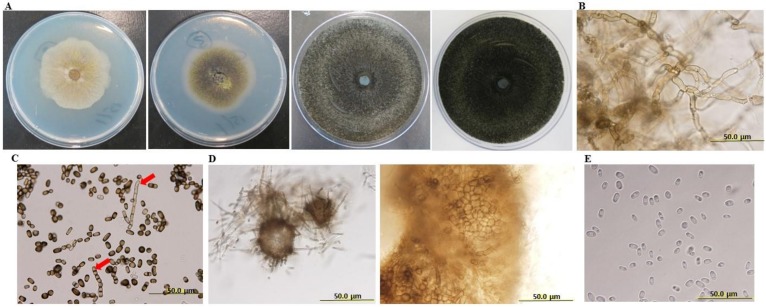
Cultural and morphological characteristics of *Neoscytalidium dimidiatum*. (**A**) Colonies on PDA (left to right: 2, 4, 8 and 12 days of incubation at 25 ± 2 °C); (**B**) mycelia; (**C**) scytalidium-like anamorph showing various shapes and maturity stages of arthroconidia (red arrows) segmenting from hyphae; (**D**) pycnidia formed on a 25-day-old colony (left) and pycnidiospores (right) on PDA; and (**E**) fusicoccum-like pycnidial conidia (immature).

First, we isolated the fungus from different symptomatic tissues on potato dextrose agar (PDA). From the cultural characteristics, the fungus grew and colonized the plate rapidly. It produced cream to white effuse, hairy to woolly colonies after 2 days of incubation (Figure 2A). The colonies turned olive green, greyish to ochraceous yellow color after 4 days. The fungus showed dark grey to black pigmentation at 8 and 12 days of incubation, respectively (Figure 2A). Microscopically, we observed mycelial growth (Figure 2B) and production of scytalidium-like anamorph of different maturity stages of arthoconidia segmenting from the hyphae (Figure 2C). We also noted that various conidial shapes ranging from ellipsoid to ovoid, rod shaped or round shaped, to hyaline with an acutely rounded apex, truncate base. Conidia were initially aseptate and brownish; at maturity, 0- to 2-septate, central cells were darker than the end cells, measuring 11.02 ± 0.33 x 4.98 ± 0.41 µm (Figure 2C). Conidiogenous cells, or pycnidial anamorph, were described as hyaline and intermingled with paraphyses, forming pycnidiospores after 25 days of incubation (Figure 2D). Cultures also produced fusicoccum-like conidia in pycnidia (Figure 2E). Together, the cultural and morphological characteristics suggest that this fungal isolate may belong to *Neoscytalidium* spp. [25]. Thus, molecular characteristics can identify the fungal specimen at the species level.

**Figure 3:**
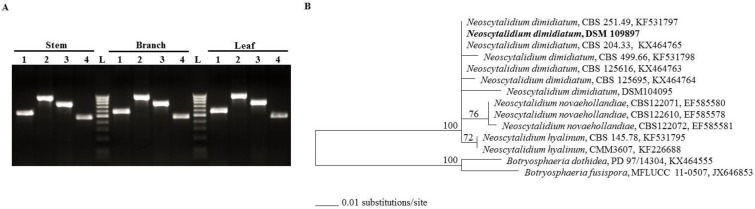
Molecular identification of *Neoscytalidium dimidiatum.* PCR amplification of specific genomic DNA regions of infected stem, branch and leaf tissues (**A**); and dendrogram showing phylogenetic relationships among *N. dimidiatum* (DSM 109897) identified in this study and other members of *Neoscytalidium* spp. prepared by the Maximum Likelihood (ML) method (**B**). In (**A**), lanes 1-4 correspond to amplifications of *ITS*, 28S rDNA region, *TEF1-α* and *β-tubulin*, respectively, in trunk (stem), branches and leaves. In (**B**), the ML tree was obtained from *TEFα-1* sequence data. The specimens used in this study carry GenBank accession number, *N. dimidiatum TEF1-α* (MN447201). Numbers at the nodes are bootstrap values after 100 replicates are expressed as percentages (LnL = −603.684353). Only values above 70% are indicated. The scale bar on the rooted tree indicates a 0.01 substitution per nucleotide position. The strain of *N. dimidiatum* from this report is indicated in bold. *Botryosphaeria dothidea*, PD 97/14304 (KX464555) and *B. fusispora* MFLUCC 11-0507 (JX646853) were used as outgroups. *ITS*, *internal transcribed spacer*; 28S rDNA, large subunit (LSU) of rDNA; *TEF1-α*, *translational elongation factor 1-α*; L, DNA ladder.

DNA-based methods are widely used to detect and identify plant pathogens. First, we isolated the fungal DNA from the PDA-grown mycelium from each tissue (stems, branches and leaves) sample. Polymerase chain reaction (PCR) amplification using primers targeting the genomic regions of *internal transcribed spacer* (*ITS*), 28S rDNA region, *translational elongation factor 1-α* (*TEF1-α*) and *β-tubulin* was performed. The amplification product of all tested genes was clearly generated in all tested specimens (Figure 3A). Because there was no available DNA sequences about the strain isolated from the UAE, the *ITS* and *TEF1-α* genes [26] were further sequenced. Sequences obtained from *ITS/LSU* rDNA and *TEF1-α* genes were also deposited in GenBank under the accession number, MN371844 and MN447201, respectively. Our data suggest that *Neoscytalidium* spp. is probably the potential fungal pathogen commonly associated with stem canker disease symptoms on royal poinciana trees.

Second, a phylogenic tree using the obtained *TEF1-α* sequence was compared to other closely related sequences in order to determine the relationship with closely *TEF1-α* related sequences coming from other *Neoscytalidium* spp. The *TEF1-α* sequence of the strain isolated from the UAE grouped in a clade representing *N. dimidiatum* (Penz.) Crous & Slippers [27] (Figure 3B). Results of the Maximum Likelihood (ML) tree indicated that the isolate, in the current study, showed >99% identity with the other isolates of *N. dimidiatum*. These isolates have been collected from different plant species such as *Juglan regia* (CBS 251.49), *Prunus* sp. (CBS 204.33), pacific madrone (*Arbutus menziesii*; CBS 204.33 and CBS 499.66), mango (*Megnifera indica*; CBS 499.66) and others (CBS 125616, CBS 125695 and DSM 104095). The identified fungal species and the other *N. dimidiatum* separately clustered from the two other species of Botryosphaeriaceae, *N. novaehollandiae* and *N. hyalinum*; thus, this isolate was identified as *N. dimidiatum*. Together, this suggests that *N. dimidiatum* (DSM 109897) is most likely the causal species of stem canker disease on royal poinciana.

### 2.3. Pathogenicity Tests of Neoscytalidium Dimidiatum on Royal Poinciana Seedlings and Apple Fruits

**Figure 4:**
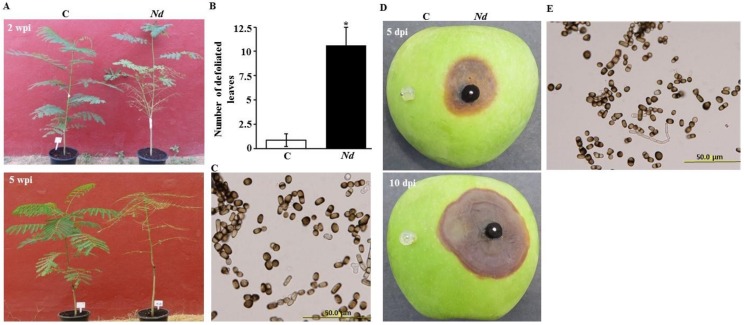
Development of canker on royal poinciana seedlings and apple fruits following artificial inoculation with *Neoscytalidium dimidiatum.* Pathogenicity test on royal poinciana seedlings inoculated (*Nd*; right) and non-inoculated (C; left) with *N. dimidiatum* at (**A**) 2 and 5 wpi; (**B**) number of defoliated leaves of inoculated and control seedlings; and (**C**) conidia after re-isolation of the pathogen from colonized stem tissues, at 5 wpi. Pathogenicity tests on (**D**) inoculated (right) and non-inoculated (left) apple fruits at 5 and 10 dpi; and (**E**) conidia of the pathogen from the inoculated apple fruits at 10 dpi. In (**B**), mean values followed by an asterisk are significantly different from control treatment at the tested time (*p* < 0.05). Experiments were repeated at least three times with similar results. C, control (no *N. dimidiatum*); *Nd*, *N. dimidiatum*.; dpi/wpi, days/weeks post inoculation.

Disease progress on one-year-old royal poinciana seedlings inoculated with 8-mm mycelial discs from 10-day-old pure culture of *N. dimidiatum* growing on PDA was regularly monitored in the greenhouse. Based on artificial inoculations, pathogenicity tests led to the development of disease symptoms on royal poinciana seedlings (Figure 4A–C). Typical symptoms of stem canker developed at the point of inoculation on the stem on plants following *N. dimidiatum* infection. At 2 weeks post inoculation (wpi), dark brown lesions formed on the surface of the stem, leaves became pale, turned yellowish in color and dropped off (Figure 4A). The disease progressed upward along the stem with black, necrotic lesions appeared at the site of inoculation; subsequently the infected stem rotted at 5 wpi. In addition, a general dryness in the plant was recognized forcing the leaves to fall (Figure 4B). In contrast, no symptoms were noticed in control seedlings. The pathogen was consistently re-isolated from all inoculated tissues and identified by conidial morphology, fulfilling Koch′s postulates (Figure 4C).

Under laboratory conditions, apple fruits were also inoculated with the same pathogen. At 5 days post inoculation (dpi), we observed discoloration of apple tissues which expanded slowly underneath the PDA plugs containing the pathogen (Figure 4D). After 10 dpi, the fungus grew into apple tissues causing rapid spreading water-soaked lesions. By peeling away the skin from the discolored tissue and placing it on PDA Petri dishes, pure cultures recovered and conidia of *N. dimidiatum* were re-isolated (Figure 4E). No disease symptoms were evident on the same apple fruit under the control plug without the pathogen at 5 and 10 dpi (Figure 4D). Altogether, disease symptoms associated with the inoculated royal poinciana seedlings and apple fruits suggest that the Koch’s postulates are fulfilled and that *N. dimidiatum* is most likely the causal agent of the stem canker disease on royal poinciana.

### 2.4. In Vitro Evaluation of Chemical Fungicides to Neoscytalidium Dimidiatum

**Figure 5:**
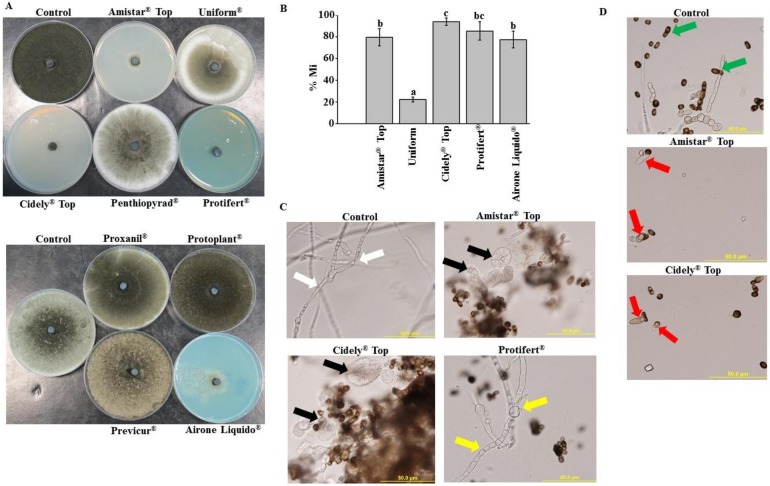
In vitro efficacy of fungicides against *Neoscytalidium dimidiatum*. (**A**) Effect of the fungicides Amistar Top^®^, Uniform^®^, Cidely^®^ Top, Penthiopyrad^®^, Protifert^®^ (top panel), Proxanil^®^, Proplant^®^, Previcur^®^ and Airone Liquido^®^ (bottom panel) at the concentration of 250 ppm on in vitro mycelial growth; and (**B**) growth inhibition rate (% Mi) of *N. dimidiatum* using 250 ppm of the fungicides after 5 days. (**C**) Abnormalities in hyphal morphology, septum formation and cytoplasmic contents; and (**D**) deformation of conidia of *N. dimidiatum* following Amistar Top^®^ and Cidely^®^ Top treatments compared to control. In (**B**)**,** values with different letters are significantly different from each other at *p* < 0.05; In (**C**)**,** white arrows indicate normal septate hyphal growth; black arrows indicate formation of non-septate hyphal formation and cytoplasmic coagulation; yellow arrows indicate lysis of hyphal wall and cytoplasm leakage. In (**D**), green arrows indicate normal formation of conidia and arthroconidia segmenting from hyphae; and red arrows indicate deformation of conidia and absence of arthroconidia.

To determine their effects on the mycelial growth of *N. dimidiatum*, PDA plates containing a final concentration of 0, 250, 500 and 1000 ppm of the chemical fungicides -available in the market- were evaluated in vitro (Figure S1). In general, we noticed varied response of *N. dimidiatum* to the selected fungicides. For example, application of the fungicides, Penthiopyrad^®^, Proxanil^®^, Protoplant^®^ and Previcur^®^ at 250 ppm (the lowest tested concentration) showed minimal or no effect on the mycelial growth of the fungus (Figure 5A). When the chemical fungicides Amistar Top^®^, Uniform^®^, Cidely^®^ Top, Protifert^®^ and Airone Liquido^®^ were, however, supplied in PDA medium, there was greater inhibition in the mycelial growth of *N. dimidiatum* at all the concentrations examined in vitro (Figure S1) including the concentration of 250 ppm (Figure 5A). These promising fungicides were also statistically (*p* < 0.05) assessed at the concentration of 250 ppm for their efficacy to inhibit the growth of *N. dimidiatum in vitro*. Among the five fungicides, medium containing a final concentration of 250 ppm of either Cidely^®^ Top or Protifert^®^ demonstrated more than 85% inhibition in growth of *N. dimidiatum*, indicating that both fungicides were considered the most efficient fungicides (Figure 5B). Although the growth inhibition rate (M%) of *N. dimidiatum* at 5 dpi reached to 77–79% after the application of Amistar Top^®^ and Airone Liquido^®^ fungicides, Uniform^®^ showed the lowest zone of inhibition (22%). This suggests that the latter fungicide is the least efficient; and therefore it is eliminated from further experiments.

We also examined the fungal pathogen microscopically in order to figure out the mode of action of the effective fungicides against *N. dimidiatum*. Results revealed that three fungicides caused significant alternations in the fungal morphology. In comparison to control treatment without any fungicide, application of either Amistar Top^®^ or Cidely^®^ Top at 250 ppm concentration to cultures led to lysis in hyphal wall and leakage in cytoplasm of *N. dimidiatum* (Figure 5C). We also noticed that Airone Liquido^®^ caused not only unusual morphological abnormalities in cultures, but also septal defects and cytoplasmic deformations in hyphal cells. Surprisingly, we observed normal, septate hyphal morphology in cultures containing Protifert^®^ similar to those in control treatment.

*N. dimidiatum* produced not only reduced numbers of deformed conidia, but also absences of arthroconidia in Amistar Top^®^- or Cidely^®^ Top-treated cultures (Figure 5D). Similar to control, cultures of Airone Liquido and Protifert^®^ showed normal conidial formation and well-formed arthroconidial segmentation produced by hyphae of *N. dimidiatum*. Altogether, the chemicals, Amistar Top^®^, Cidely^®^ Top and Airone Liquido, had a direct effects on *N. dimidiatum* DSM 109897 through the inhibition of mycelial growth and induction of morphological abnormalities; thus, the former two fungicides shared a common mechanism of action. The mode of action of Protifert^®^ in competently inhibiting the mycelial growth of *N. dimidiatum* was not determined. Because there are many reports in which chemical control against plant pathogens has proven successful only under laboratory conditions, more reliable in vivo studies are needed for the reproducibility of the results obtained from those of in vitro testing.

### 2.5. Assessment of Chemical Fungicides on Neoscytalidium Dimidiatum Using Apple Bioassay

**Figure 6:**
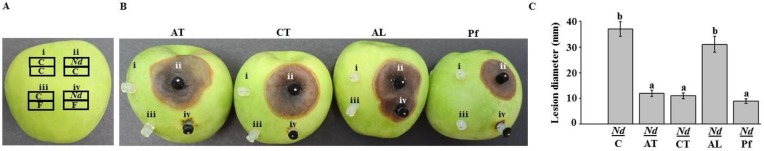
In vivo inhibitory effect of the chemical fungicides against *Neoscytalidium dimidiatum* using the “apple fruit bioassay”. An illustration showing (**A**) inoculated-apple fruit with the chemical fungicides and/or *N. dimidiatum* agar plugs in combinations; (**B**) apple fruit bioassays using chemical fungicides; and (**C**) lesion diameter of *N. dimidiatum* using 250 ppm of the fungicides after 10 dpi. In (**A–B**), (i) two sterile non-inoculated PDA agar plugs; (ii) *N. dimidiatum* inoculum alone with a sterile agar plug below it; (iii) the fungicide (F) alone with a sterile agar plug above it; and (iv) pairing *N. dimidiatum* and the fungicide together, with the fungicide on the apple surface and *N. dimidiatum*-inoculated plug on top of the fungicide. In (**C**), values with different letters are significantly different from each other at *p* < 0.05. C, control (no *N. dimidiatum*); *Nd*, *N. dimidiatum;* F, fungicide; AT, Amistar Top^®^, CT, Cidely^®^ Top; AL, Airone Liquido^®^; Pf, Protifert^®^; dpi, days post inoculation.

To evaluate the most effective fungicides against *N. dimidiatum*, we developed the apple fruit bioassay method (Figure 6A). Placing the pathogen alone on apple fruits resulted in relatively large-sized, brown-colored lesions with distinct edges (Figure 6B). In contrast, none of the fungicides tested had negative effects against the pathogen. Excluding Airone Liquido^®^, when a plug containing any of the three fungicides paired with a plug of *N. dimidiatum* on the surface of the fruit, the particular fungicide completely suppressed the pathogen and no lesions were formed compared to the pathogen treatment alone (Figure 6B).

The fungicides Amistar Top^®^, Cidely^®^ Top and Protifert^®^ caused significantly (*p* < 0.05) smaller lesion sizes than the positive control (*N. dimidiatum*) treatment (Figure 6C). However, we did not notice any significant (*p* < 0.05) difference between the treatments of Airone Liquido^®^ and the pathogen alone. Therefore, Airone Liquido^®^ was excluded from further experiments. To greater extent, three chemical fungicides completely prevented lesion development on apple fruits. Overall, the novel apple fruit bioassay led to the selection of three prominent fungicides, Amistar Top^®^, Cidely^®^ Top and Protifert^®^, which could have the potential to manage stem canker disease on royal poinciana seedlings.

### 2.6. Fungicide Effects on Royal Poinciana Infected with Neoscytalidium Dimidiatum

**Figure 7:**
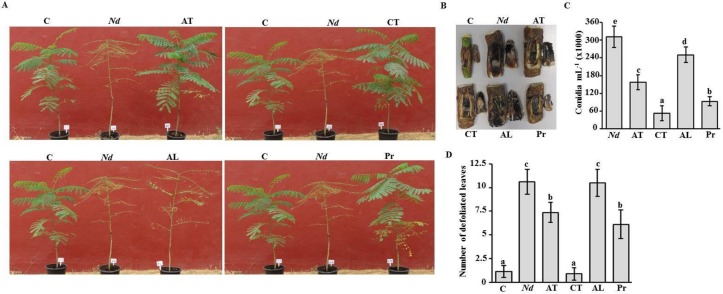
Effect of fungicide treatments on artificially inoculated royal poinciana seedlings with *Neoscytalidium dimidiatum* in the greenhouse. Fungicidal suppression of stem canker disease on royal poinciana seedlings using (**A**) potential chemical fungicides; (**B**) symptoms of inoculated regions; (**C**) number of conidia after recovery of the pathogen from stem tissues; and (**D**) number of defoliated leaves in inoculated seedlings sprayed with chemical fungicides at 4 wpt. In (**A–D**), seedlings were inoculated for 2 weeks with *N. dimidiatum* before the fungicide treatment. In (**C & D**), mean values with different letters are significantly different from each other at *p* < 0.05. Experiments were repeated at least three times with similar results. C, control (no *N. dimidiatum*); *Nd*, *N. dimidiatum*; AT, Amistar Top^®^: CT, Cidely^®^ Top; AL, Airone Liquido^®^, Pr, Protifert^®^; wpt, weeks post treatment.

In the greenhouse experiment, we tested the efficacy of the most promising fungicides at 4 weeks post treatment (wpt) on *N. dimidiatum*-inoculated royal poinciana plants. Seedlings were artificially inoculated with the fungal pathogen for 2 weeks when symptoms of stem canker disease were easily recognized (Figure S2). Diseased plants were treated with a particular fungicide and this treatment was considered as 0 wpt. Disease progress or plant recovery of fungicide-treated plants was monitored until the end of the evaluation period of 4 wpt. In general, *N. dimidiatum*-inoculated plants that were sprayed with water only showed stem canker disease symptoms such as drying branches, falling leaves and discoloring stems, resulting in almost completely bare seedlings (Figure 7A). This was also clear in the longitudinal wood necrosis in these diseased plants (Figure 7B). In contrast, inoculated plants that were treated with Amistar Top^®^, Cidely^®^ Top or Protifert^®^ fungicide clearly showed vegetative growth recovery (Figure 7A) and developed relatively healthy wood (Figure 7B) at 4 wpt comparable to the negative control plants (no prior artificial infection). Affected plants treated with Airone Liquido^®^ showed similar disease symptoms as diseased plants (Figure 7A). Peeling away the periderm of the inoculated plants that were treated with Airone Liquido^®^ revealed the presence of a black layer of fungal growth from which *N. dimidiatum* was reisolated (Figure 7B).

The effects of each of the chemical fungicides were also determined according to the number of conidia progressing on diseased- and treated-seedlings. In general, there was a significant (*p* < 0.05) difference between all treatments (Figure 7C). This was accompanied with a dramatic decrease in the number of conidia in Cidely^®^ Top-treated seedlings that nearly reached to 6-fold reduction compared to that of untreated plants. We noticed that the number of conidia of *N. dimidiatum* recovered from the stems of royal poinciana treated with Protifert^®^ and Amistar Top^®^ fungicides was 3.3- and 2-fold less than in the control, respectively (Figure 7C). Airone Liquido^®^ was marked the least spore counts; and thus it was considered the least effective among all tested fungicides.

The number of defoliated leaves was also assessed on diseased- and recovered-seedlings as an indication on the severity of disease symptoms on seedlings at 4 wpt. Based on our results, Cidely^®^ Top treatment was comparable to the treatment without inoculation (Figure 7D). This was evident by the similar number of defoliated leaves per plant. On the other hand, the same plants showed significantly (*p* < 0.05) less falling leaves than inoculated-seedlings without fungicide treatment at the same period of evaluation. At 4 wpt, defoliated leaves demonstrated 31–42% reduction on seedlings sprayed with Amistar Top^®^ and Protifert^®^, respectively, in comparison to *N. dimidiatum*-inoculated seedlings without any fungicide treatment (Figure 7D). It was also clear that Airone Liquido^®^ was not efficient enough, confirming our previous results on the number of conidia recovered from inoculated seedlings using the same fungicide. Our data imply that Cidely^®^ Top seems to be the most effective fungicide because the severity of stem canker disease is gradually suppressed and the pathogen is more or less restrained.

### 2.7. Effect of Cidely® Top on Royal Poinciana Trees Naturally Infected with Neoscytalidium Dimidiatum

**Figure 8:**
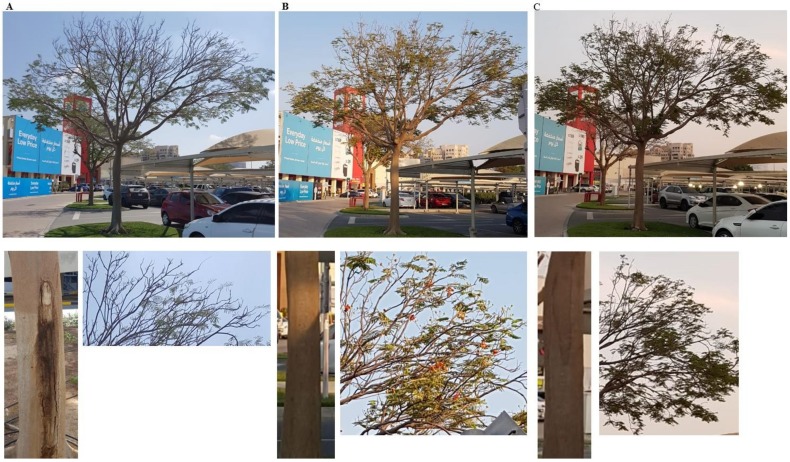
Effect of Cidely^®^ Top on royal poinciana trees naturally infected with *N. dimidiatum* in the field in DFC, UAE. Fungicidal suppression of stem canker disease symptoms on (**A**) royal poinciana trees (*n* = 6); followed by treatment with the fungicide Cidely^®^ Top at (**A**) 0 (**B**) 16 and (**C**) 32 weeks post treatment. In (**A–C**), photos showed the severe disease symptoms and the recovery of the same whole tree (upper panel), trunk (left, bottom panel) and branches (right, bottom panel).

We confirmed the results obtained from the in vitro and in vivo experiments by applying the promising fungicide Cidely^®^ Top on royal poinciana trees naturally affected by stem canker under field conditions. Royal poinciana trees were sprayed with 250 ppm of Cidely^®^ Top, and severity of symptoms or recovery of the trees was monitored for 32 weeks. Typical disease symptoms were observed on the day of fungicidal application (0 wpt; Figure 8A). After 16 weeks of spraying with Cidely^®^ Top, disease severity was remarkably decreased in the treated trees (Figure 8B). This was evident by diminishing trunk damage and developing new fresh shoots. It was also noted that trees treated with Cidely^®^ Top fungicide increased their vegetative growth and were completely recovered at 32 wpt (Figure 8C). This suggests that the application with Cidely^®^ Top results in disappearance of disease symptoms, ultimately leading to nice looking, healthy trees.

## 3. Discussion

Royal poinciana is a large deciduous tree species prevalent in subtropical and tropical areas of the world. It is valued as a local street tree and is widely planted in open areas [1]. In the last decade, this beautiful flowering plant has become widespread in urban and agricultural areas of the UAE. Although it is known for its ability to withstand severe conditions, diseases are major factors that affect the health of royal poinciana [3,4]. Many of the phytopathogens can cause diseases on host plants, including royal poinciana [5,6,7,8]. Therefore, careful attention should be attained to the causal agent of stem canker disease on royal poinciana, taking into account the frequency of disease incidence, the geographical distribution and the environmental conditions favorable to the disease occurrence.

In our efforts to identify the pathogen linked with the diseased trees, we first detected the symptoms of stem canker on royal poinciana. In general, we noticed dieback, canker and gummosis, which ultimately led to complete dryness and death of royal poinciana trees (Figure 1). Although some studies have reported several fungi to cause cankers on plant species [5,6,7,8], others have recorded *N. dimidiatum* on almond, dragon fruit, eucalyptus, fig and plum, displaying disease symptoms of canker and dieback in different places of the world [13,14,15,28]. In general, environmental stress has negative impact on the severity of disease, depending on the level and duration of the stress, and the sensitivity and developmental stage of the plant species. In hot summers, sooty canker invades trees and ornamentals of mulberry, ash, walnut, fig, sycamore, apple, apricot, poplar, eucalyptus and olive in Iraq [29,30]. In Oman, significant damage due to dieback, witling and death of royal poinciana has been reported to be caused by *N. dimidiatum* and symptoms are even worsened when trees are exposed to heat (up to 45 °C) and shortage of water [16]. All previously mentioned reports are in agreement with the findings of the current study. Yet, there are no reports about the causal agent of the disease symptoms of stem canker on royal poinciana or any other ornamental woody tree in the UAE. Previously, the fungal pathogens *T. punctulata* and *F. solani* have been shown to cause black scorch disease and SDS on date palm, respectively [17,19,31] and *L. theobromae* to cause dieback disease on mango [18]. Therefore, accurate fungal identification was carried out, along with proper chemical fungicide treatment to manage the devastating damage of this disease on royal poinciana.

The fungal pathogen was constantly isolated from all symptomatic tissues examined from trees of royal poinciana, and it was characterized based on its morphology, phylogeny and pathogenicity assays. On PDA, a rapid growth of mycelia filling the entire plate was observed within 8 days. The culture was effuse, hairy to wooly, started as white with creamy, ochraceous-yellowish color that turned to dark greyish or blackish color by day 12. Similar observations have been previously reported on *N. dimidiatum* isolated from diseased trees of eucalyptus [23]. Microscopic examination of the pathogen demonstrated branched and septate hyphae with no conidiophores. Consistent with [21], arthroconidia were thick-walled and barrel-shaped that could be found individually or in chains, ranging 5–15 x 3–6 µm in size (Figure 2). Old cultures, of 25 days, developed hyaline pycnidial conidia when young, and dark brown central regions when aged. Cultures also produced fusicoccum-like conidia in pycnidia (Figure 2) [32]. Because *Neoscytalidium* spp. are very close and difficult to discriminate, molecular characterization was followed to avoid misleading conclusions about the pathogen. For that reason, phylogenetic analysis using *TEF1-α* sequence (MN447201) was generated and proved the identity of the fungus as *N. dimidiatum*. *N. dimidiatum* was closely related to both *N. novaehollandiae* [33] and *N. hyalinum* [34], confirming previous findings [13,35]. Our data indicated that the isolate of *N. dimidiatum* in the current study was morphologically and genetically similar to other isolates of *N. dimidiatum* from *Juglan regia*, *Prunus* sp., mango and others. Therefore, isolate DSM 109897 in the present study belonged to *N. dimidiatum* and was the main causal agent of stem canker on royal poinciana in the UAE. Our observations on the symptoms and the pathogen associated with stem canker disease on royal poinciana are similar to a previous report on the same tree in Oman [16]. This suggests that *N. dimidiatum* may possibly have been introduced from this neighboring country to the UAE.

The existence of the pathogen and the progression of the disease in tissues of the whole royal poinciana seedlings and apple fruits were further verified via pathogenicity tests. The results obtained from the greenhouse experiment on young healthy plants after inoculation were similar to the disease symptoms on trees of royal poinciana located in the field, and that was confirmed by Koch’s postulates when *N. dimidiatum* was frequently recovered from the inoculated seedlings. Our data match those in other trials using artificial inoculation of the same pathogen on royal poinciana [16] or other plant species [13,14,15,20,28]. Pathogenicity assays on seedlings of royal poinciana (Figure 4), *F. benjamina* and *F. nitida* [13] and eucalyptus, poplar and olive [30] clearly described that discoloration of vascular tissues, and drying and defoliation of leaves, were symptoms associated with stem canker caused by *N. dimidiatum*. There has been a rise in reports about *N. dimidiatum* causing diseases on fruits of pitahaya, plum and almond [12,14,15]. Apple fruit bioassays have been conducted to determine the effects of the fungal pathogen associated with canker diseases [5,36]. Therefore, we performed pathogenicity tests on healthy apple fruits and monitored the disease progress.

There are some examples of using BCAs effective against *N. dimidiatum* or other pathogens [37,38,39]; yet these studies have not been assessed *in vivo*. For example, *Trichoderma harzianum* T3.13 revealed in vitro antagonistic activities to *N. dimidiatum* [39]. Although chemical fungicides have adverse effect on human health, food and environment [23,40], these agents are commonly used due to their relatively low cost, rapid acting, long lasting, high stability and ease of application [41]. Under laboratory conditions, four of the tested chemicals, Amistar Top^®^, Cidely^®^ Top, Protifert^®^ and Airone Liquido^®^, showed suppression in the growth of *N. dimidiatum*. This was evidenced by the abnormalities seen in hyphal morphology, septal formation, cytoplasmic contents and the deformation of conidia following fungicide treatments (Figure 5). Previously, Cidely^®^ Top exhibited the strongest inhibition of mycelial growth of *T. punctulata* and *L. theobromae* in petri dish experiments [17,42]. The same fungicides were further evaluated in vivo using apple fruit bioassay (Figure 6). In general, Amistar Top^®^, Cidely^®^ Top and Protifert^®^, significantly reduced the lesion size on apple fruits when 250 ppm of the fungicide was applied concurrently with the pathogen. On contrast, Airone Liquido^®^ was not effective against this pathogen on apple and was carried out in further experiments as a negative control. We claim that the novel apple bioassay is a small-scale reference of what may occur in the greenhouse/field. In vivo experiments using carrot roots and mango fruits have previously been implemented to assess growth retardation of *Pythium coloratum* and L. theobromae by BCAs, respectively [22,43].

Recent reports have shown that in vitro tests along with greenhouse experiments are essential to determine the sensitivity of plant pathogens to chemical and/or biological treatments [17,18,19,22,42]. According to our greenhouse experiments, Cidely^®^ Top, followed by Protifert^®^ and then Airone Liquido^®^ were effective on diseased seedlings of royal poinciana. It is known that the organic foliar fertilizer, Protifert^®^, is a good source of minerals, essential traces, amino acids and peptides necessary for plant growth and development. In this study, we also showed that Protifert^®^ not only provided vigorous and healthy seedlings, but also it served as a protection to trees from fungal infections i.e., *N. dimidiatum*. Under greenhouse conditions, we noticed that the most significant reduction in disease symptoms of stem canker was found in Cidely^®^ Top-treated seedlings of royal poinciana at 4 wpt. This was clear in seedlings sprayed with Cidely^®^ Top possessing the lowest conidial counts and the least number of defoliated leaves, indicating that this fungicide could be a potent fungicide for the management of *N. dimidiatum* affecting royal poinciana trees. The result of Cidely^®^ Top is in agreement with previous studies indicating high effectiveness of this fungicide against a number of fungal pathogens attacking trees such as *T. punctulata*, *L. theobromae* and *F. solani* that were almost completely inhibited [19,22,42]. To a lesser extent, Amistar Top^®^ was not as effective as Protifert^®^ or Cidely^®^ Top in reducing the pathogenic activities of *N. dimidiatum* in greenhouse trials. Eventhough Amistar Top^®^ and Cidely^®^ Top were difenoconazole-based fungicides sharing the same concentration of the active ingredient; the superior efficiency of Cidely^®^ Top over Amistar Top^®^ could be attributed to the presence of cyflufenamid as an additional active ingredient leading to increased inhibition levels of *N. dimidiatum*. Difenoconazole was ineffective against *Fusarium magniferae* [44], but it was significantly capable for managing other diseases [17,18,42,45,46], including stem canker on royal poinciana in the current study (Figure 7). This can be disputed to the fungicide application methods, active ingredient concentrations, plant growth conditions or pathogen responses. Airone Liquido^®^ (metal copper), on the other hand, is not recommended to manage the disease. 

So far, there are no reports to evaluate Cidely^®^ Top or any systemic fungicide on royal poinciana trees infected with *N. dimidiatum* under field conditions. Thus, the same fungicide was found to be highly effective against plant pathogenic fungi on date palm and mango [18,19,42]. Accordingly, a field experiment was carried out to assess the efficacy of Cidely^®^ Top on naturally infested royal poinciana plants. Apparently, the entire trees showed “more or less” full recovery that was mainly observed in newly developed inflorescences (branches with flower clusters) and reduced disease symptoms on trunks of royal poinciana trees sprayed with Cidely^®^ Top at 16 and 32 wpt (Figure 8). This suggests that Cidely^®^ Top can possibly serve as a competent element of IDM of stem canker on royal poinciana. Here, we report the symptoms, the pathogen as well as the proper chemical treatment to manage stem canker as the first step toward planning IDM programs against this devastating disease on royal poinciana in the UAE or elsewhere. In the current study, the phenotype *i.e.,* symptoms associated with the disease can be considered as a starting point for future comparative ‘omic’ analyses including genomes and responses to environmental variation [47]. A combination of different methods to achieve suitable IDM practices is on top of our priorities. Investigations for cultural (pruning), chemical (Cidely^®^ Top and Protifert^®^) and BCAs as IDM to manage stem canker on royal poinciana are in progress for environmental sustainability.

## 4. Materials and Methods

### 4.1. Fungal Culture and Isolation

Eight-year-old royal poinciana trees located in DFC, Dubai, UAE (latitude/longitude: 25.22/55.36) were associated with longitudinal cankers on stems (Figure 1). Cross-sections in trunks and branches were made and drying leaves were gathered from diseased trees. All collected tissues were then transferred to the Plant Microbiology Laboratory, Department of Biology, United Arab Emirates University in Al Ain city, UAE, for isolation and identification purposes. To isolate the pathogen, affected tissues were cut into small pieces (3–5 mm long), washed and surface-sterilized with mercuric chloride 0.1% for 1 min, and 1.05% NaOCl for 5 min; followed by three consecutive washings in sterile distilled water. They were then transferred onto PDA (Lab M Limited, Lancashire, UK) plates, supplemented with 25 mg/L penicillin-streptomycin (Sigma-Aldrich Chemie GmbH, Taufkirchen, Germany) to inhibit bacterial contaminants. Petri dishes were incubated for 5 days at 25 ± 2 °C. Once grown out of the plated tissue, mycelia were aseptically sub-cultured on fresh PDA and purified using hyphal-tip isolation technique [48]. To characterize fungal structures, mycelia and conidia were observed using Nikon-Eclipse 50i light microscope (Nikon Instruments Inc., Melville, NY, USA). The culture of the identified fungus, *N. dimidiatum* [27], was deposited in Leibniz-Institute DSMZ-German Collection of Microorganisms and Cell Cultures GmbH (Braunschweig, Germany) under the accession number 109897.

### 4.2. Molecular Identification of the Pathogen

DNA of the pathogen isolated from diseased of stem, branch and leaf tissues was extracted from mycelia cultured for 10 d at 25 °C on PDA plates, using the fungi DNA isolation kit (Norgen Biotek Corp., Thorold, ON, Canada). PCR was set up to amplify target regions of internal transcribed spacer (*ITS*) of the nuclear rDNA for *N. dimidiatum* using ITS1 and ITS4 primers [26], partial *28S rDNA* using LR0R and LR5 primers [49], partial *TEF1-α* using EF1-728F and EF1-986R [50] and partial *β-tubulin* using Bt1a and Bt1b [51]. PCR reactions (50 µL) contained 30-ng DNA template, 50 pmol of each primer, 200 µM of each dNTP, 2.5 unit of Taq DNA polymerase and 2.2 mM buffer (MgCl_2_). Each cycle of PCR was set as the following: 94 °C for 1 min; 58 °C for 1 min; and 72 °C for 1 min (total of 32 cycles). All primer sequences can be found in Table S1. All protocols for amplification and sequencing were as described [26].

The sequence of *TEF1-α* gene of the fungal isolate from the UAE was deposited in GenBank (accession number: MN447201). The phylogenetic tree using *TEF1-α* sequence, obtained from DSMZ, was constructed against other sequences of *TEF1-α* belonging to *Neoscytalidium* spp. [27] retrieved from GenBank-NCBI (www.ncbi.nlm.nih.gov). ML analysis was performed for the estimation of the phylogenetic tree [52] after all sequences were aligned. Phylogenetic trees were validated with a statistical support of the branches with 100 bootstrap resamples. The following isolates used in the analysis belong to *N. dimidiatum, N. novaehollandiae, N. hyalinum, Botryosphaeria dothidea* and *B. fusispora*.

### 4.3. In Vivo Pathogenicity Tests and Koch’s Postulates

Pathogenicity tests were conducted on one-year-old healthy royal poinciana seedlings (*n* = 9), purchased from the local market. Using sterile scalpels, the bark of the main stem was wounded and inoculations under the wounded bark were performed at 30–50 cm above the soil surface [13]. An agar plug (8-mm-diameter) colonized by mycelium of 10-day-old culture of *N. dimidiatum* was placed into the wound, where the mycelium facing inner parts, and wrapped using parafilm. Control royal poinciana seedlings were inoculated with sterile agar plugs (no pathogen). Plants were maintained in the greenhouse (15 h day/9 h night at 25 ± 2 °C) and were evaluated for symptoms and disease progression at 2 and 5 wpi. By the end of the experiment, the fungus was re-isolated from the point of infection on PDA and compared morphologically with the inoculated fungus. 

Disease was assayed on disease-free apple fruits (cv Granny Smith), purchased from local fresh markets, to find out the effect of *N. dimidiatum*. Fruits (*n* = 8) were washed with sterile distilled water, surface-sterilized with 70% ethanol and wounded with a sterilized scalpel (2 mm diameter) according to [36] with some modifications. On each fruit, one agar plug (11 mm in diameter) containing mycelium of *N. dimidiatum* (colonized mycelium facing down) and one agar control plug without pathogen was applied. Inoculated fruits were maintained in dark (at 25 ± 2 °C and 80% relative humidity) and lesion size was rated for an interval of 5 d for 10 d. At 10 dpi, pieces from regions showing disease symptoms of inoculated fruit tissues were removed, surface sterilized, plated and incubated, as mentioned above. Structures of conidia and mycelium were morphologically compared with the inoculated fungus.

### 4.4. In Vitro Evaluation of Fungicides Against N. Dimidiatum

The fungicide experiment was carried out according to the previously described procedures [17,18,19]. The selected fungicides along with their active ingredients can be found in Table S2. Fungal growth was assessed on each fungicide with a final concentration of 0 (control), 250, 500, 750 and 1000 ppm aseptically introduced into sterilized PDA plates, supplied with penicillin-streptomycin antibiotics, at 25 ± 2 °C. The tested fungal pathogen was introduced to PDA plates using a sterile cork-borer (8 mm diameter). Cultures were incubated at 25 ± 2 °C for 10 days, and percentage of the mycelial growth inhibition was measured according to:

% Mi = (Mc – Mt)/Mc × 100%(1)

where Mi, inhibition of the mycelial growth; Mc, colony diameter (in mm) of control set; and Mt; colony diameter (in mm) of the target fungus on the medium with fungicide.

### 4.5. In Vivo Evaluation of Selected Fungicides

To determine the ability of fungicides to reduce lesion formation after *N. dimidiatum* inoculation under laboratory conditions, an apple fruit bioassay was developed. The apple fruit bioassay was modified according to previous bioassays on carrot and mango against *Pythium coloratum* and *L. theobromae*, respectively [18,22,43]. Healthy apple fruits (cv. Granny Smith) were washed with sterile distilled water, surface-sterilized with 70% ethanol and placed in plastic trays on wet, sterile filter papers. Apple fruits were then inoculated using agar plugs (11 mm) colonized by the selected fungicide and/or *N. dimidiatum*, as described above, onto each apple fruit according to the following combinations: (i) two sterile non-inoculated PDA agar plug (control; C); (ii) *N. dimidiatum* alone with a sterile PDA agar plug below it; (iii) the fungicide alone with a sterile PDA agar plug above it; and (iv) pairing *N. dimidiatum* and the fungicide together (the fungicide on the apple surface and *N. dimidiatum*-inoculated plug on top of the fungicide). All fungicides were introduced onto the apple surface 24 h before inoculation with the pathogen to have enough time for the active ingredients to disperse uniformly onto the apple surface. Each apple fruit was inoculated with the four combinations for each fungicide of five fruits/tray and was replicated three times. Trays were covered with aluminum foil and incubated in dark (at 25 ± 2 °C and 80% relative humidity) for 10 d. Lesion diameters were measured (in mm) and averaged.

In a greenhouse experiment, we assessed the impact of each fungicide on one-year-old royal poinciana seedlings. Seedlings were wounded and inoculated with agar plugs containing mycelium of *N. dimidiatum* in the stem of each plant as described above. Inoculated plants were maintained in the greenhouse at 25 °C until symptoms were evident. At 2 wpi, seedlings were either sprayed with 250 ppm fungicide or water (control); and these treatments were designated as 0 wpt. Symptoms on inoculated plants, conidia counts of the fungal pathogen and the number of falling leaves were recorded at 4 wpt [42]. The procedure of conidia counts involved homogenized weight of affected tissues placed in 5 mL of water, and the suspended material was assessed to estimate the number of conidia using haemocytometer (Agar Scientific Limited, Essex, UK).

Regarding the field experiments, trees were located in the same place described above. Cidely^®^ Top (Syngenta International AG, Basel, Switzerland) was the only tested fungicide on six royal poinciana trees (8 years old). Each *N. dimidiatum* naturally infested tree was chosen so as to be surrounded by untreated corresponding trees to serve as a reservoir for recontamination. Trees were pruned and completely sprayed/treated with the recommended dose of the fungicide (250 ppm). Experiments were repeated twice in February 2018 and February 2019 with similar results.

### 4.6. Statistical Analysis

For the pathogenicity assays, fruits (*n* = 5) and seedlings (*n* = 9) for each treatment were used. For the in vitro evaluation of fungicides against *N. dimidiatum*, 6 plates for each treatment were used. For the fungal conidia counts and the number of falling leaves in the in vivo evaluation of fungicides under greenhouse conditions, a minimum of 4 plants for each treatment was used. Data represent the mean ± SD. Analysis of Variance (ANOVA) and Duncan’s multiple range test were performed to determine the statistical significance at *p* < 0.05. All experiments were independently repeated three times with similar results. All statistical analyses were performed by using SAS Software version 9 (SAS Institute Inc., Cary, NC, USA).

## Supplementary Materials

Supplementary materials can be found at https://www.mdpi.com/1422-0067/21/3/1033/s1.

## References

[1] Gledhill D. (2008). The Names of Plants.

[2] Kirtikar K.R., Basu B.D. (1999). Indian medicinal plants.

[3] Gilman E.F., Watson D.G., Klein R.W., Koeser A.K., Hilbert D.R., McLean D.C. (2019). Delonix regia: Royal Poinciana.

[4] Cayley D.M. (1923). Fungi associated with “die back” in stone fruit trees. Ann. Appl. Biol..

[5] Anagnostakis S.L., Ferrandino F.J. (1998). Isolation of *Nectria galligena* from cankers on sweet birch. Plant Dis..

[6] Rouxel T., Balesdent M.H. (2005). The stem canker (blackleg) fungus, *Leptosphaeria maculans*, enters the genomic era. Mol. Plant Pathol..

[7] Chungu D., Muimba-Kankolongo A., Wingfield M.J., Roux J. (2010). Identification of fungal pathogens occurring in eucalypt and pine plantations in Zambia by comparing DNA sequences. Forestry.

[8] Darge W.A. (2017). First report of *Lasioplodia theobromae* causing needle blight and stem canker diseases on *Araucaria heterophylla* in Ethiopia. J. Hortic. Res..

[9] Reckhaus P. (1987). Hendersonula dieback of mango in Niger. Plant Dis..

[10] Farr D.F., Bills G.F., Chamuris G.P., Rossman A.Y. (1989). Fungi on Plants and Plant Products in the United States.

[11] Ray J.D., Burgess T., Lanoiselet V.M. (2010). First record of *Neoscytalidium dimidiatum* and *N. novaehollandiae* on *Mangifera indica* and *N. dimidiatum* on *Ficus carica* in Australia. Australas. Plant Dis. Notes.

[12] Yi R.H., Lin Q.L., Mo J.J., Wu F.F., Chen J. (2015). Fruit internal brown rot caused by *Neoscytalidium dimidiatum* on pitahaya in Guangdong province, China. Australas. Plant Dis. Notes.

[13] Al-Bedak O.A., Mohamed R.A., Seddek N.H. (2017). First detection of *Neoscytalidium dimidiatum* associated with canker disease in Egyptian *Ficus* trees. Forest Pathol..

[14] Hajlaoui M.R., Nouri M.T., Hamrouni N., Trouillas F.P., Ben Yahmed N., Eddouzi J., Mnari-Hattab M. (2018). First record ofdieback and decline of plum caused by *Neoscytalidium dimidiatum* in Tunisia. New Dis. Rep..

[15] Nouri M.T., Lawrence D.P., Yaghmour M.A., Michailides T.J., Trouillas F.P. (2018). *Neoscytalidium dimidiatum* causing canker, shoot blight and fruit rot of almond in California. Plant Dis..

[16] Elshafie A.E., Ba-Omar T. (2002). First report of *Albizia lebbeck* caused by *Scytalidium dimidiatum* in Oman. Mycopathologia.

[17] Saeed E.E., Sham A., El-Tarabily K.A., Abu Elsamen F., Iratni R., AbuQamar S.F. (2016). Chemical control of dieback disease on date palm caused by the fungal pathogen, *Thielaviopsis punctulata*, in United Arab Emirates. Plant Dis..

[18] Saeed E.E., Sham A., AbuZarqa A., Al Shurafa K., Al Naqbi T.S., Iratni R., El-Tarabily K.A., AbuQamar S.F. (2017). Detection and management of mango dieback disease in the United Arab Emirates. Int. J. Mol. Sci..

[19] Alwahshi K.J., Saeed E.E., Sham A., Alblooshi A.A., Alblooshi M.M., El-Tarabily K.A., AbuQamar S.F. (2019). Molecular identification and disease management of date palm sudden decline syndrome in the United Arab Emirates. Int. J. Mol. Sci..

[20] Chuang M.F., Ni H.F., Yang H.R., Shu S.L., Lai S.Y., Jiang Y.L. (2012). First report of stem canker disease of pitaya (*Hylocereus undatus* and *H. polyrhizus*) caused by *Neoscytalidium dimidiatum* in Taiwan. Plant Dis..

[21] Mohd M.H., Salleh B., Zakaria L. (2013). Identification and molecular characterizations of *Neoscytalidium dimidiatum* causing stem canker of red-fleshed dragon fruit (*Hylocereus polyrhizus*) in Malaysia. J. Phytopathol..

[22] Kamil F.H., Saeed E.E., El-Tarabily K.A., AbuQamar S.F. (2018). Biological control of mango dieback disease caused by *Lasiodiplodia theobromae* using streptomycete and non-streptomycete actinobacteria in the United Arab Emirates. Front Microbiol..

[23] Al-Tememe Z.A.M., Lahuf A., Abdalmoohsin R.G., Al-Amirry A.T. (2019). Occurrence, identification, pathogenicity and control of *Neoscytalidium dimidiatum* fungus, the causal agent of sooty canker on *Eucalyptus camaldulensis* in Kerbala Province of Iraq. 2019. Plant Arch..

[24] Murad N.Y., Al-Dabagh M.N. (2014). Evaluation some of pesticides in control of *Neoscytalidium dimidiatum* (Penz) Crous and Slippers causing wilt and canker on cypress trees in Iraq. Iraqi J. Agric. Sci..

[25] Farr D.F., Elliott M., Rossman A.Y., Edmonds R.L. (2005). *Fusicoccum arbuti* sp. nov. causing cankers on pacific madrone in western North America with notes on *Fusicoccum dimidiatum*, the correct name for *Scytalidium dimidiatum* and *Nattrassia mangiferae*. Mycologia.

[26] White T.J., Bruns T., Lee S., Taylor J. (1990). Amplification and direct sequencing of fungal ribosomal RNA genes for phylogenetics. PCR Protoc..

[27] Crous P.W., Slippers B., Wingfield M.J., Rheeder J., Marasas W.F.O., Philips A.J.L., Alves A., Burgess T., Barber P., Groenewald J.Z. (2006). Phylogenetic lineages in the Botryosphaeriaceae. Stud. Mycol..

[28] Du B.D., Ngoc D.T.B., Thang N.D., Tuan L.N.A., Thach B.D., Hien N.Q. (2019). Synthesis and in vitro antifungal efficiency of alginate-stabilized Cu_2_O-Cu nanoparticles against *Neoscytalidium dimidiatum* causing brown spot disease on dragon fruit plants (*Hylocereus undatus*). Vietnam J. Chem..

[29] Hassan W.A., Pasha A.A., Mohammad M.B. (2009). Sooty canker on some thin bark trees caused by *Nattrassia mangiferae*. Egypt. J. Agric. Res..

[30] Hassan W.A., Haleem R.A., Hassan P.H. (2011). Effect of heat-stress predisposition on the development of sooty canker caused by *Neoscytalidium dimidiatum* (Penz.) Crous & Slippers. Acta Agrobot..

[31] Alhammadi M.S., Al-Shariqi R., Maharachchikumbura S., Al-Sadi A.M. (2018). Molecular identification of fungal pathogens associated with date palm root diseases in the United Arab Emirates. J. Plant Pathol..

[32] Pavlic D., Wingfield M.J., Barber P., Slippers B., Hardy G.E.S., Burgess T.I. (2008). Seven new species of the Botryosphaeriaceae from baobab and other native trees in Western Australia. Mycologia.

[33] Polizzi G., Aiello D., Vitale A., Giuffrida F., Groenewald Z., Crous P.W. (2009). (2009) First report of shoot blight, canker, and gumumosis caused by *Neoscytalidium dimidiatum* on citrus in Italy. Plant Dis..

[34] Madrid H., Ruı´z-Cendoya M., Cano J., Stchigel A., Orofino R., Guarro J. (2009). Genotyping and in vitro antifungal susceptibility of *Neoscytalidium dimidiatum* isolates from different origins. Int. J. Antimicrob. Agents.

[35] Alwan S.L., Hussein H.N. (2019). Efficacy of ecofriendly biocontrol *Azotobacter chroococcum* and *Lactobacillus rhamnosus* for enhancing plant growth and reducing infection by *Neoscytalidium* spp. in fig (*Ficus carica* L.) saplings. J. Kerbala Agric. Sci..

[36] Hortova B., Novotny D., Erban T. (2014). Physiological characteristics and pathogenicity of eight *Neofabraea* isolates from apples in Czechia. Europ. J. Hort. Sci..

[37] AbuQamar S., Moustafa K., Tran L.-S. (2017). Mechanisms and strategies of plant defense against *Botrytis cinerea*. Crit. Rev. Biotechnol..

[38] Mengiste T., Laluk K., AbuQamar S., Prusky D., Gullino M.L. (2010). Mechanisms of induced resistance against B. cinerea. Post-harvest Pathology.

[39] Rusmarini W., Shah U.K.D., Abdullah M.P., Mamat S., Hun T.G. (2017). Identification of *Trichoderma harzianum* T3.13 and its interaction with *Neoscytalidium dimidiatum* U1, a pathogenic fungus islated from dragon fruit (*Hylocereus polyrhizus*) in Malaysia. Int. J. Environ. Agric. Res..

[40] Budzinski H., Couderchet M. (2018). Environmental and human health issues related to pesticides: From usage and environmental fate to impact. Environ. Sci. Pollut. Res..

[41] Kuai X., Barraco C., Després C. (2017). Combining fungicides and prospective NPR1-based “just-in-time” immunomodulating chemistries for crop protection. Front. Plant Sci..

[42] Saeed E.E., Sham A., Salmin Z., Abdelmowla Y., Iratni R., El-Tarabily K.A., AbuQamar S.F. (2017). *Streptomyces globosus* UAE1, a potential effective biocontrol agent for black scorch disease in date palm plantations. Front. Microbiol..

[43] El-Tarabily K.A., Hardy G.E.St.J., Sivasithamparam K., Hussein A.M., Kurtböke D.I. (1997). The potential for the biological control of cavity spot disease of carrots caused by *Pythium coloratum* by streptomycete and non-streptomycete actinomycetes in Western Australia. New Phytol..

[44] Iqbal Z., Pervez M.A., Ahmad S., Iftikhar Y., Yasin M., Nawaz A., Ghazanfar M.U., Dasti A.A., Saleem A. (2010). Determination of minimum inhibitory concentrations of fungicides against fungus *Fusarium mangiferae*. Pak. J. Bot..

[45] Khan S.H., Idrees M., Muhammad F., Mahmood A., Zaidi S.H. (2004). Incidence of shisham (*Dalbergia sissoo* Roxb.) decline and in vitro response of isolated fungus spp. to various fungicides. Int. J. Agric. Biol..

[46] Yanase Y., Katsuta H., Tomiya K., Enomoto M., Sakamoto O. (2013). Development of a novel fungicide, penthiopyrad. J. Pestic. Sci..

[47] AbuQamar S.F., Moustafa K., Tran L.S. (2016). ‘Omics’ and plant responses to *Botrytis cinerea*. Front. Plant Sci..

[48] Kirsop B.E., Doyle A. (1991). Maintenance of microorganisms and cultured cells, a manual of laboratory methods.

[49] Vilgalys R., Hester M. (1990). Rapid genetic identification and mapping of enzymatically amplifed ribosomal DNA from several *Cryptococcus* species. J. Bacteriol..

[50] Carbone I., Kohn L.M. (1999). A method for designing primer sets for speciation studies in filamentous ascomycetes. Mycologia.

[51] Glass N.L., Donaldson G.C. (1995). Development of primer sets designed for use with the PCR to amplify conserved genes from filamentous Ascomycetes. Appl. Environ. Microbiol..

[52] Tamura K., Stecher G., Peterson D., Filipski A., Kumar S. (2013). MEGA6: Molecular evolutionary genetics analysis version 6.0. Mol. Biol. Evol..

