# Supplementary material for: Molecular Characterization and Disease Control of Stem Canker on Royal Poinciana (Delonix regia) Caused by Neoscytalidium dimidiatum in the United Arab Emirates
Source: Int J Mol Sci. 2020 Feb 4;21(3):1033. doi: 10.3390/ijms21031033 (PMC7036867; doi:10.3390/ijms21031033)
Supplement: Supplementary file 1 [file ijms-21-01033-s001.pdf]

Supplementary Materials: Molecular Characterization and Disease Control of Stem Canker on Royal Poinciana (*Delonix regia*) Caused by *Neoscytalidium dimidiatum* in the United Arab Emirates

Seham M. Al Raish, Esam Eldin Saeed, Arjun Sham, Khulood Alblooshi, Khaled A. El-Tarabily and Synan F. AbuQamar

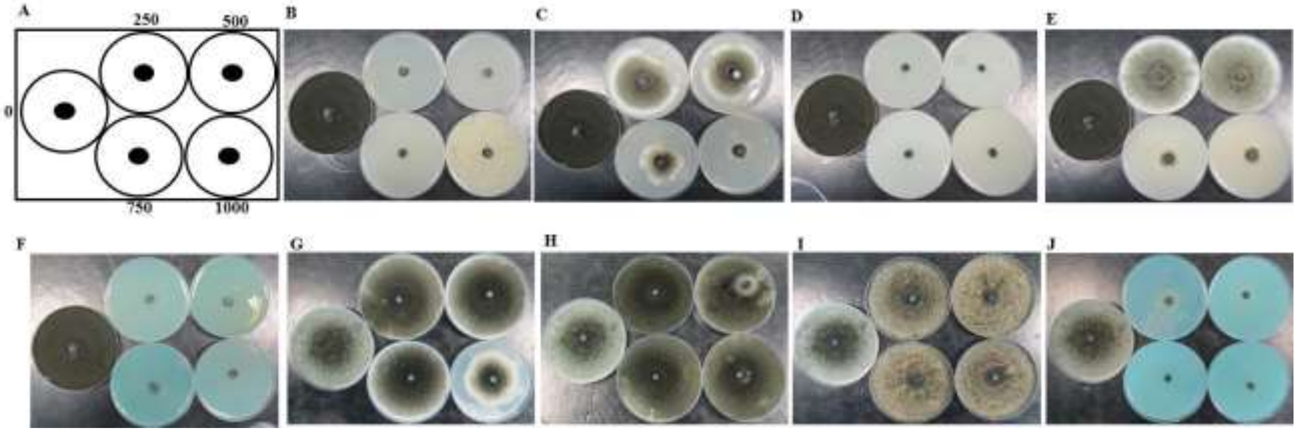

Figure S1. Growth inhibition effect of fungicides on *Neoscytalidium dimidiatum*. (A) An illustration showing inoculated-PDA plates containing different concentrations (in ppm) with the colonized *N. dimidiatum* agar plugs. Growth inhibitory effect on *Neoscytalidium dimidiatum* using the fungicides: (B) Amistar® Top; (C) Uniform®; (D) Cidely® Top; (E) Penthiopyrad®; (F) Protifert®; (G) Proxanil®; (H) Proplant®; (I) Previcur®; and (J) Airone Liquido® on PDA pales. In (A), black circles represent colonized *N. dimidiatum* agar plugs. In (B-J), photos were collected 5 days after inoculation.

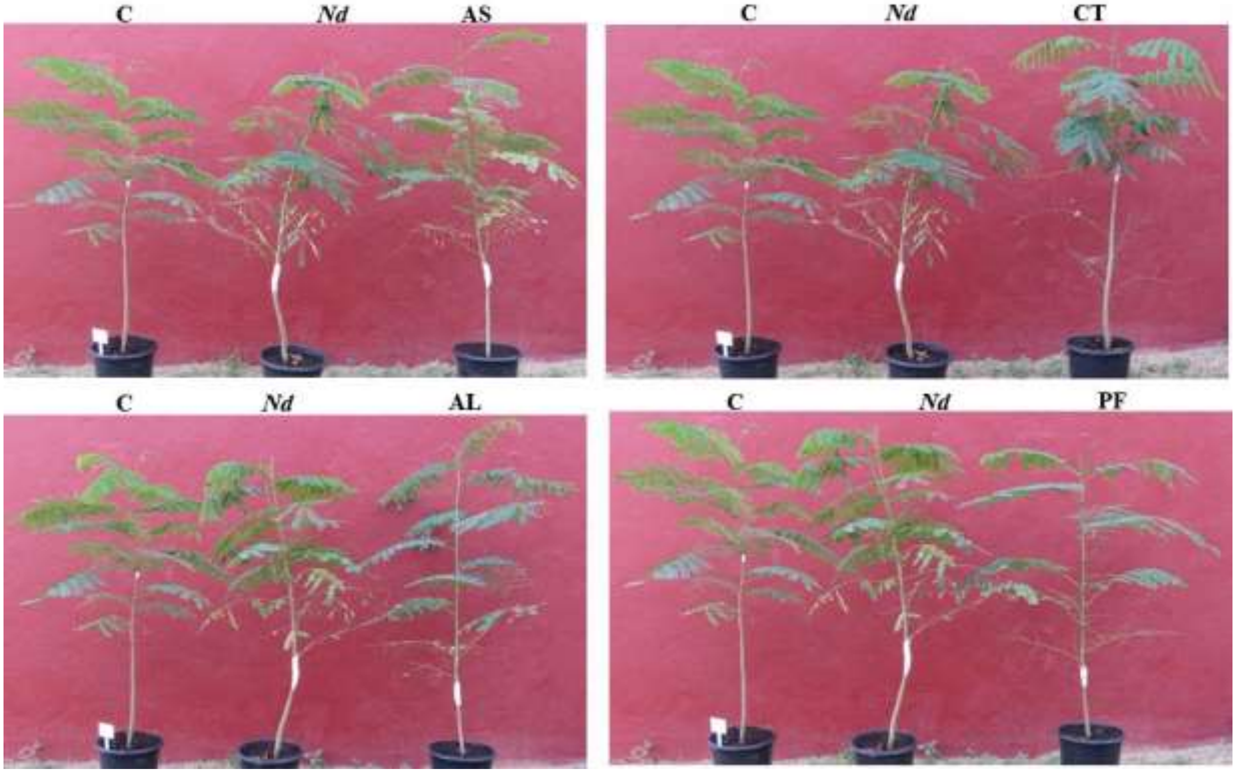

Figure S2. Stem canker disease symptoms on royal poinciana seedlings before the fungicide treatment (0 wpt; 2 wpi with *N. dimidiatum*. C, control (no *N. dimidiatum*); *Nd*, *N. dimidiatum*; AT, Amistar® Top, CT, Cidely® Top; Pr, Protifert®; AL, Airone Liquido®; wpt/wpi, weeks post treatment/inoculation.

Table S1. List of PCR primers (sequence 5′ to 3′) used in this study.

Description	Left primer sequence	Right primer sequence
ITS	ITS1: TCCGTAGGTGAACCTGCGG	ITS4: TCCTCCGCTTATTGATATGC
28S DNA	LR0R: ACCCGCTGACTTAAGC	LR5: TCCTGAGGGAAACTTCG
B-tubulin	Bt1a: TTCCCCCGTCTCCACTTCTTCATG	Bt1b: GACGAGATCGTTCATGTTGAACTC
TEF1-α	EF1F: TGCGGTGGTATCGACAAGCGT	EF2R: AGCATGTTGTCGCCGTTGAAG

Table S2. Labels, active ingredients, producing company and country of the fungicides used in this study.

Fungicide	Active ingredients	Company	Country
Amistar Top®	Azoxystrobin 200 g/L + Difenoconazole 125 g/L	Syngenta	Switzerland
Uniform®	Azoxystrobin 322 g/L + Metalaxyl-M 124 g/L		
Cidely® Top	Difenoconazole 125 g/L + Ciflufenamide 15 g/L		
Protifert®	Copper sulfate pentahydrate 20% + Aminoacides 19% + Sulfur 8% + Total nitrogen 3.3%	Sicit 2000 S.P.A.	Italy
Airone Liquido®	Metal copper 272 g/L	Gowan Italia S.P.A.	
Proxanil®	Propamocarb hydrochloride 400 g/L + Cymoxanil 50 g/L	Arysta LifeScience	Belgium
Protoplant®	Propamocarb 722 g/L		
Penthiopyrad®	Penthiopyrad 40%	Mitsui Chemicals Agro Inc.	Japan
Previcur®	Propamocarb hydrochloride 530 g/L + Fosetyl-aluminium 310 g/L	Bayer	Germany
